# Bioresorbable vascular scaffolds: between promises and reality

**DOI:** 10.18632/oncotarget.20630

**Published:** 2017-09-01

**Authors:** Rocco A. Montone, Adam J. Brown, Giampaolo Niccoli

**Affiliations:** Rocco A. Montone: Department of Cardiovascular and Thoracic Sciences, Catholic University of the Sacred Heart, Rome, Italy and Interventional Cardiology, ASL Viterbo, Belcolle Hospital, Viterbo, Italy

**Keywords:** bioresorbable vascular scaffolds, outcome, stent thrombosis

Bioresorbable vascular scaffolds (BRS) have been developed in the hope of improving the clinical outcomes over existing metallic dug-eluting stents (DES). Potential limitations of DES include the permanent presence of a metallic foreign body within the artery and a durable polymer, either of which may cause vascular inflammation, neoatherosclerosis, restenosis and perpetuate the risk of very-late stent thrombosis (ST). Moreover, permanent metallic stents indefinitely impair physiological vasomotor function and preclude against late luminal enlargement and the potential for future bypass grafting within the stented segment [1]. The introduction of BRS held promise in addressing some of these issues. BRS aim to provide transient scaffolding of the vessel, preventing acute vessel closure/recoil and then disappear over a period of time. Drug elution by BRS prevents neointimal proliferation in similar fashion to DES, and complete bioresorption is associated with late vessel lumen enlargement, plaque regression, and restoration of vasomotion. Hence, BRS hold the potential to finally achieve the paradigm of vascular restoration therapy, facilitating both arterial remodelling and vascular function [1].

The Absorb BVS (Abbott Vascular, Santa Clara, CA, USA; hereafter referred to as BVS), is a 150 μm thick bioresorbable poly(l-lactide) everolimus eluting scaffold [1]. BVS obtained FDA approval for US marketing and have been widely implanted around the world, with more than 100,000 BVS deployed. Their use was based on findings from randomised clinical trials demonstrating non-significant differences in 1-year outcomes between patients who received BVS compared with metallic everolimus-eluting stent (EES) [2, 3]. However, 2-year data deriving from ABSORB III (Ellis S. Everolimus-eluting Bioresorbable Vascular Scaffolds in Patients with Coronary Artery Disease: ABSORB III Trial 2-Year Results. ACC Conference, Washington, 18 March 2017) and AIDA [4] raised safety concerns on medium-long term outcome of BVS.

Recently, we published a meta-analysis of randomised trials with a follow up of 2-year or longer assessing medium-long term safety and efficacy of BVS versus EES [5]. We included seven trials, comprising data for 5,583 patients randomised to receive either BVS (*n* = 3,261) or EES (*n* = 2,322). Importantly, patients treated with BVS had a higher risk of definite/probable ST compared with patients treated with EES (OR 3.33 [95% CI 1.97-5.62], *p* < 0.00001). BVS patients also had a higher risk of subacute (between 24 hours and 30 days), late (between 30 days and 1 year) and very-late ST (>1 year), whereas the risk of acute ST (< 24 hours) was similar. Moreover, patients treated with BVS compared with EES had a higher risk at 2-year of target-lesion failure (TLF) (OR 1.47 [95% CI 1.14-1.90], *p* = 0.003), mainly driven by an increased risk of target-vessel myocardial infarction (OR 1.73 [95% CI 1.31-2.28], *p* = 0.0001; *I*^2^ = 0%), and of target-lesion revascularization (TLR) (OR 1.27 [95% CI 1.00-1.62], *p* = 0.05). The risk of TLF and TLR for BVS patients was higher between year 1 and 2, whereas there was no difference in the first year. Finally, the risk of cardiac death was similar between the groups.

Accordingly, the US Food and Drug Administration (FDA) issued a safety alert warning physicians that treating patients with the BVS may increase the risk of major cardiac events. In Europe the manufacturer restricted the use of BVS to centres participating in clinical trials or registries.

Whether the risk of scaffold thrombosis can be mitigated by a specific implantation technique (e.g. mandatory pre-dilatation, accurate vessel sizing and high-pressure post-dilatation), by avoiding implantation in small arteries, or by prolonging dual antiplatelet therapy is subject to debate. With respect to the implantation technique, post-hoc analyses have suggested that this may mitigate some of the risk, but these technical factors have not yet been prospectively validated [6]. It is also increasingly recognised that strut thickness, which is about two times higher (150 µm vs approximately 80 µm) with the BVS than with new-generation DES, may adversely affect coronary flow and shear forces, precipitating platelet activation and even scaffold thrombosis. Long-term dual antiplatelet therapy up to the time of complete resorption may be a reasonable precautionary measure as long as the individual risk of bleeding is carefully considered.

Although high standards have been set by newer-generation DES, there are still several unmet clinical needs that may favour use of BRS technologies (i.e. treatment of diffuse disease, procedures in young individuals, restoration of vascular physiology). Device improvements in new-generation DES successfully addressed issues observed with early-generation devices, and it is highly likely that further device iterations of BVS will overcome the current issue of scaffold failure. Of note, Magmaris magnesium BRS (Biotronik, Germany) showed favourable early clinical outcomes and an experimental study revealed a reduced thrombogenicity compared with Absorb BVS [7] in the porcine model. However, larger clinical studies are needed in order to confirm safety and efficacy of this device.
